# DDIT4 promotes gastric cancer proliferation and tumorigenesis through the p53 and MAPK pathways

**DOI:** 10.1186/s40880-018-0315-y

**Published:** 2018-07-05

**Authors:** Feng Du, Lina Sun, Yi Chu, Tingyu Li, Chao Lei, Xin Wang, Mingzuo Jiang, Yali Min, Yuanyuan Lu, Xiaodi Zhao, Yongzhan Nie, Daiming Fan

**Affiliations:** State Key Laboratory of Cancer Biology, National Clinical Research Center for Digestive Diseases and Xijing Hospital of Digestive Diseases, Fourth Military Medical University, 127 Chang Le West Road, Xi’an, 710032 China

**Keywords:** DNA damage-inducible transcript 4, Gastric cancer, Proliferation, Mitogen-activated protein kinase, p53

## Abstract

**Background:**

Gastric cancer (GC) is one of the most common malignancies worldwide, particularly in China. DNA damage-inducible transcript 4 (DDIT4) is a mammalian target of rapamycin inhibitor and is induced by various cellular stresses; however, its critical role in GC remains poorly understood. The present study aimed to investigate the potential relationship and the underlying mechanism between DDIT4 and GC development.

**Methods:**

We used western blotting, real-time polymerase chain reaction, and immunohistochemical or immunofluorescence to determine DDIT4 expression in GC cells and tissues. High-content screening, cell counting kit-8 assays, colony formation, and in vivo tumorigenesis assays were performed to evaluate cell proliferation. Flow cytometry was used to investigate cell apoptosis and cell cycle distribution.

**Results:**

DDIT4 was upregulated in GC cells and tissue. Furthermore, downregulating DDIT4 in GC cells inhibited proliferation both in vitro and in vivo and increased 5-fluorouracil-induced apoptosis and cell cycle arrest. In contrast, ectopic expression of DDIT4 in normal gastric epithelial cells promoted proliferation and attenuated chemosensitivity. Further analysis indicated that the mitogen-activated protein kinase and p53 signaling pathways were involved in the suppression of proliferation, and increased chemosensitivity upon DDIT4 downregulation.

**Conclusion:**

DDIT4 promotes GC proliferation and tumorigenesis, providing new insights into the role of DDIT4 in the tumorigenesis of human GC.

**Electronic supplementary material:**

The online version of this article (10.1186/s40880-018-0315-y) contains supplementary material, which is available to authorized users.

## Background

Gastric cancer (GC) is one of the most common malignancies worldwide and remains the second leading cause of cancer-related death in China [1]. Despite the declining incidence of GC, the 5-year overall survival rate remains low at less than 30% [2]. Two of the major causes of the poor prognosis of GC are metastasis and multiple drug resistance, which greatly hamper the success of treatment modalities [1]. Genetic alterations are generally considered to drive cancer development and progression, and emerging evidence indicates that the complex molecular heterogeneity of GC underlies most, if not all, of the general ineffectiveness of current chemotherapeutics for GC [3]. Hence, improvements in the treatment of GC must be developed from a better understanding of its elaborate regulatory network because the underlying molecular mechanisms of GC tumorigenesis remain unclear.

DNA damage-inducible transcript 4 (DDIT4), also known as DNA damage response 1 (REDD1) and stress-triggered protein (RTP801), is induced by a variety of stress conditions, including oxidative stress, endoplasmic reticulum stress, hypoxia, and starvation [4]. DDIT4 protein inhibits mammalian target of rapamycin complex 1 (mTORC1) by stabilizing the tuberous sclerosis complex (TSC1–TSC2) [5]. Over the past decades, DDIT4 dysregulation has been observed in numerous human malignancies. In prostate cancer (PC) cells, DDIT4 enhances CCAAT/enhancer-binding protein beta (C/EBPbeta)-mediated autophagosome-lysosome fusion and desensitizes the cells to bortezomib [6]. Additionally, baicalein upregulates DDIT4 and inhibits mTORC1 and the proliferation of platinum-resistant cancer cells, indicating that DDIT4 expression has potential as a chemotherapeutic and chemoprevention agent [7]. Although mammalian target of rapamycin (mTOR) pathway inhibition is a current strategy for the treatment of cancer [8, 9], paradoxically, several in vitro and in vivo studies indicate that DDIT4 has a protective role against apoptosis [10–12]; *DDIT4* knockdown increases dexamethasone-induced cell death in murine lymphocytes [10]. Additionally, DDIT4 expression was significantly increased in serous adenocarcinoma compared with other histological types, and this increase was positively associated with ascites formation and late-stage disease in ovarian cancer (OC) [11]. A recent in silico evaluation of the online datasets Kaplan–Meier plotter and SurvExpress indicated that high DDIT4 levels were significantly associated with a worse prognosis in acute myeloid leukemia, glioblastoma multiforme, and breast, colon, skin and lung cancer [12]. However, in GC, the second most common type of cancer in Asia in terms of incidence and cancer mortality, the clinical significance and biological role of DDIT4 remain to be elucidated.

In the present study, we examined DDIT4 expression levels in GC tissue samples and cell lines, and investigated the role of DDIT4 and the mechanism by which it is dysregulated in gastric cancer.

## Methods

### Cell culture and tissue collection

The human GC cell lines SGC7901, BGC823, MKN45, and AGS, and the immortalized gastric epithelial cell line GES were purchased from the Cell Resource Center of the Chinese Academy of Sciences, Shanghai, China. Cells were maintained in Dulbecco’s Modified Eagle’s Medium (Thermo Scientific HyClone, Beijing, China) supplemented with 10% fetal bovine serum (HyClone), 100 U/mL penicillin, and 100 U/mL streptomycin (HyClone) in a 37 °C humidified incubator with a mixture of 95% air and 5% CO_2_.

A total of 20 fresh primary GC samples and matched adjacent non-cancerous tissues were obtained from patients undergoing surgery at Xijing Hospital, Xi’an, China. All samples were confirmed by the Department of Pathology at Xijing Hospital and stored in a liquid nitrogen canister. All patients provided informed consent for excess specimens to be used for research purposes and all protocols employed in the present study were approved by the Medical Ethics Committee of Xijing Hospital.

### Mice

Female BALB/c nude mice were provided by the Experimental Animal Center of the Fourth Military Medical University and were housed in pathogen-free conditions. All animal studies complied with the Fourth Military Medical University animal use guidelines, and the protocol was approved by the Fourth Military Medical University Animal Care Committee.

### Reagent and inhibitor

5-Fluorouracil was purchased from Sigma (Sigma-Aldrich Corporation, Los Angeles, CA, USA), and MAPK/ERK inhibitor (PD98059) and p53 inhibitor (A15201) were purchased from Invitrogen (Thermo Fisher Scientific, Cambridge, Massachusetts, USA); all were stored according to the manufacturer’s instructions.

### RNA extraction and real-time polymerase chain reaction (PCR)

Total RNA was extracted from cell lines using the RNeasy Plus Universal Tissue Mini Kit (Qiagen, Hilden, Germany) according to the manufacturer’s instructions. The PCR primers for *DDIT4* and *ACTB* were synthesized by TaKaRa (Dalian, China). The sequences were as follows: *DDIT4*, 5′-GGACCAAGTGTGTTTGTTGTTTG-3′ (Forward) and 5′-CACCCACCCCTT CCTACTCTT-3′ (Reverse); *ACTB*, 5′-TCATGAAGTGTGACGTTGACATCCGT- 3′ (Forward) and 5′-CCTAGAAGCATTTGCGGTGCACGATG-3′ (Reverse). cDNA was synthesized using the PrimeScript RT Reagent Kit (TaKaRa, Dalian, China). Real-time PCR was performed using SYBR Premix Ex Taq II (TaKaRa). Fluorescence was measured using a LightCycler 480 system (Roche, Basel, Switzerland). *ACTB* was used as an internal control for mRNA analysis. Each sample was run in triplicate.

### Protein extraction and western blotting

Total proteins were prepared from fresh frozen tissue or cultured cells in radio immunoprecipitation assay (RIPA) lysis and extraction buffer (Beyotime Biotechnology, Shanghai, China) with protease and phosphatase inhibitors. Denatured proteins (20–50 mg) were separated by sodium dodecyl sulfate–polyacrylamide gel electrophoresis and transferred to polyvinylidene difluoride membranes. The following primary antibodies were used according to the manufacturer’s instructions: anti-DDIT4 (Dilution 1:500, Abcam, Cambridge, MA, USA) and anti-β-actin (Dilution 1:2000), anti-Ki67 (Dilution 1:1000), anti-p53 (Dilution 1:1000), anti-p-p53 (p-Ser6) (Dilution 1:1000), anti-p-p53 (p-Ser315) (Dilution 1:1000), anti-p21^Cip1^ (Dilution 1:500), anti-p-p21^Cip1^ (p-Thr145) (Dilution 1:500), anti-MEK1 (Dilution 1:1000), anti-p-MEK1 (p-Ser221) (Dilution 1:1000), anti-p42/44MAPK (Dilution 1:1000), and anti-p-p42/44MAPK (p-Thr202 and p-Tyr204) (Dilution 1:1000) (Cell Signaling Technology, Beverly, MA, USA). Densitometry of specific blotted bands was analyzed by ImageJ 1.48 software (Image-Processing and Analysis in Java; National Institutes of Health, Bethesda, MD, USA; http://imagej.nih.gov/), and the intensity values were normalized against the β-actin loading control.

### Tissue microarray immunohistochemistry

GC tissue microarrays were purchased from Superchip (Shanghai, China). Each array contained 90 cases of paired adjacent gastric tissues and primary GC tissues. Immunohistochemical (IHC) staining was performed using anti-DDIT4 and anti-Ki67 antibodies as per the manufacturer’s instructions. IHC results were scored independently by two pathologists in a blinded manner. The scoring was based on the intensity and extent of staining. Staining intensity was graded as follows: 0, negative staining; 1, weak staining; 2, moderate staining; and 3, strong staining. The proportion of stained cells per specimen was determined semi-quantitatively as follows: 0, < 1%; 1, 1–25%; 2, 26–50%; 3, 51–75%; and 4, > 75%. The histological score (H-score) for each specimen was computed using the following formula: H-score = proportion score×intensity score. The samples were further characterized by H-score as negative (−, score: 0), weak (+, score: 1–4), moderate (++, score: 5–8), and strong (+++, score: 9–12). Samples with an H-score of > 4 were considered to exhibit high expression, and samples with an H-score of ≤ 4 were considered to exhibit low expression.

### Immunofluorescence staining

Cells were plated onto glass coverslips, fixed with 4% paraformaldehyde for 20 min and permeabilized with 0.1% Triton X-100 in PBS for 15 min. Blocking solution was applied at room temperature for 1 h. Rabbit anti-human DDIT4 primary antibody (Abcam) was applied at 4 °C overnight. FITC-conjugated goat anti-rabbit and Cy3-conjugated goat anti-mouse secondary antibodies were incubated on the coverslips at room temperature for 2 h. Immunostaining signals and DAPI-stained nuclei were visualized at room temperature using a confocal microscope (FV10i; Olympus, Tokyo, Japan) equipped with a 10×/0.30 numerical aperture objective lens (Olympus) and Fluoview software (version 4.3; Olympus). No imaging medium was used. For better visualization, the images were adjusted using the level and brightness/contrast tools in Photoshop according to the guidelines for the presentation of digital data.

### Lentivirus infection

*DDIT4*-overexpression or sh-DDIT4 lentivirus infection was conducted by GeneChem (Shanghai, China). Target cells (1 × 10^5^) were infected with 1 × 10^7^ lentivirus-transducing units in the presence of 5 mg/mL polybrene. An empty lentiviral vector was used as a negative control. After transfection and antibiotic selection for 6 weeks, cells were collected for further investigation.

### High-content screening assay

Cells transfected with lentivirus stably expressing green fluorescent protein (GFP) were seeded into 96-well plates and treated with increasing doses of 5-FU (0, 10, 20 µg/mL for GC cells or 0, 2, 4 µg/mL for GES cells). Cells were imaged every 4 h for 48 h using the Thermo Scientific CellInsight CX7 High Content Analysis Platform (0, 10, 20 µg/mL for GC cells or 0, 2, 4 µg/mL for GES cells; Thermo Fisher Scientific, Cambridge, Massachusetts, USA). Proliferation curves were plotted and analyzed using HCS Studio Software (Thermo Fisher Scientific).

### Colony formation assays

Transfected cells were seeded in six-well plates (1000 cells/well). After 14–18 days of incubation to establish stable clones, cells were fixed with 70% ethanol and stained with crystal violet solution. Colonies containing greater than 50 cells were counted. The experiment was conducted with three independent triplicates.

### Cell cycle and apoptosis assays

For cell cycle analysis, target cells were selected with antibiotics (penicillin–streptomycin solution) for 48 h after transfection as indicated, fixed in 75% ethanol, and stained with propidium iodide supplemented with RNase A (Roche, Mannheim, Germany) for 30 min at 22 °C. The Annexin V-FITC Apoptosis Detection Kit (Cell Signaling Technology, Beverly, MA, USA) was used for apoptosis assays. Cells (1 × 10^4^) were stained according to the manufacturer’s protocol and sorted using a fluorescence-activated cell sorting sorter (BD Biosciences, La Jolla, CA, USA). Data were analyzed using ModFit software (BD Biosciences).

### In vivo tumorigenicity

BGC823 cells (5 × 10^5^ cells in 0.2 mL of PBS) transfected with pCMV-DDIT4 or empty pCMV were injected subcutaneously into the dorsal flank of 5-week-old female Balb/c nude mice (five mice per group). Tumor diameter was measured every 3 days for 30 days. Tumor volume (mm^3^) was calculated based on the longest and shortest diameters as follows: volume = (shortest diameter)^2^ × (longest diameter) × 0.5. Thirty days after injection, all mice were killed, and the tumor xenografts were isolated for further analysis. All experimental animals were supplied by the Experimental Animal Center of the Fourth Military Medical University. All protocols for animal studies were approved by the Fourth Military Medical University Animal Care Committee.

### Cell counting kit-8 (CCK8) assay

For cell counting kit 8 assays, cells were seeded into 96-well plates at a density of 1000 cells in 100 μL of complete culture medium per well. At the indicated time points, the medium was replaced with a kit solution (TransDetect cell counting kit, Transgene, Beijing, China) and complete culture medium at a ratio of 1:9, and the samples were incubated for 2 h at 37  °C. The absorbance of each sample was analyzed at 450 nm using a microtiter plate reader (Tecan, Switzerland). The assay was repeated in triplicate.

### Phospho-specific protein microarray analysis

Phospho-array detection was performed in cooperation with Wayen Biotechnology (Shanghai, China). At 48 h post-transfection, all treated cells were collected for protein extraction. Protein samples of 50 mg each were tagged with biotin reagent and hybridized on a Phosphorylation ProArray (Full Moon BioSystems, USA) using an Antibody Array Kit (FullMoon BioSystems, USA) for the detection of 248 site-specific cancer signaling phospho-antibody profiles. Finally, fluorescence intensity was scanned by GenePix 4000B (Axon Instruments, Houston, USA) using GenePix Pro 6.0. The raw data were manipulated using Grubbs’ method. The phosphorylation ratio was calculated as follows: phosphorylation ratio ¼ phospho value/unphospho value.

### Statistical analyses

SPSS software (version 19.0, SPSS Inc., Chicago, IL, USA) was used for statistical analyses. Continuous data are presented as the mean ± standard deviation (SD), and Student’s unpaired *t*-test was utilized for comparisons between two groups. Frequencies of categorical variables were compared using the χ^2^ test. A *P* value of less than 0.05 was considered significant.

## Results

### DDIT4 was upregulated in GC tissues

**Fig. 1:**
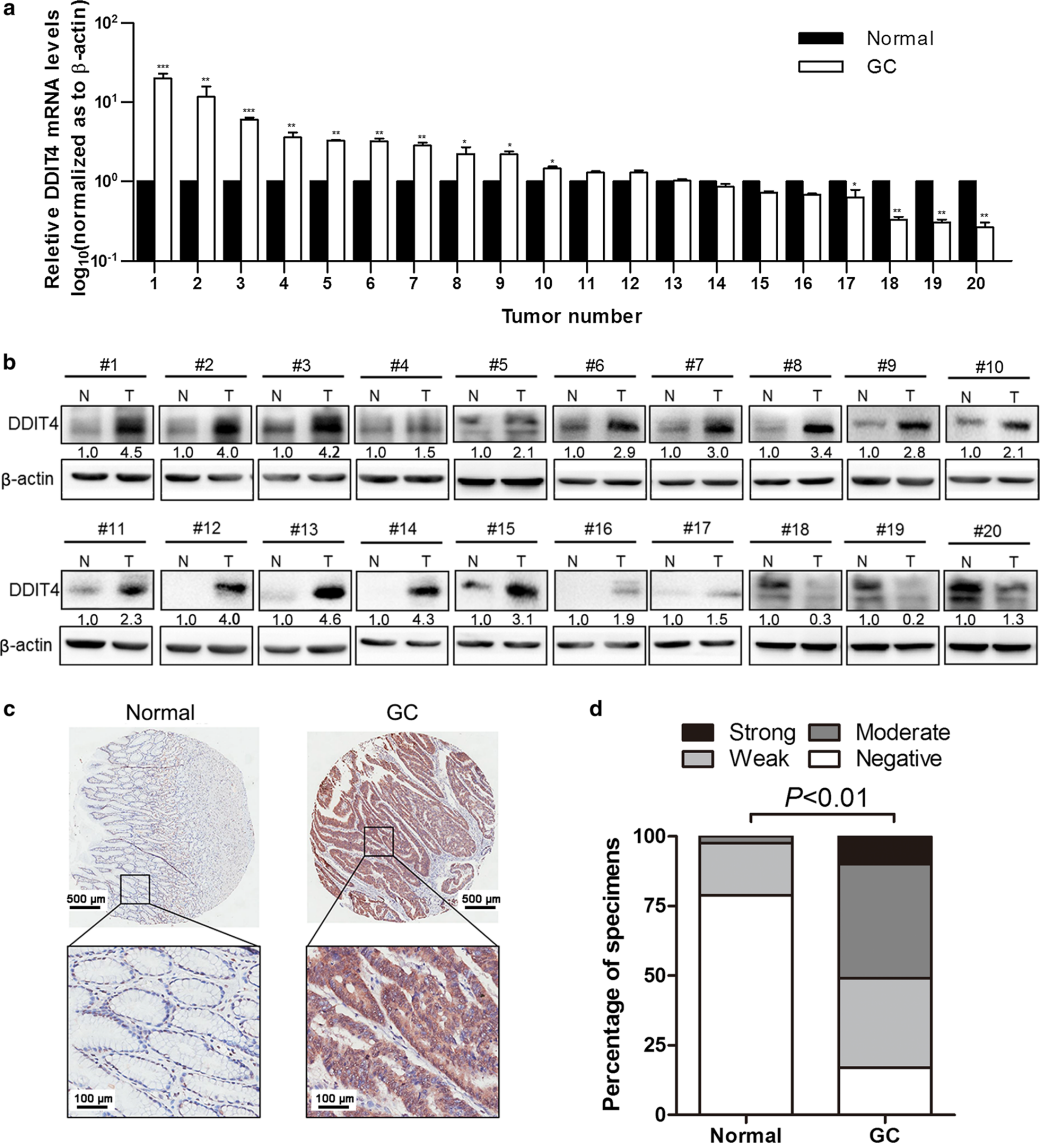
DDIT4 expression levels in GC tissues and adjacent normal tissues. **a** Relative *DDIT4* levels normalized to *ACTB* levels in GC tissues and adjacent normal tissues were detected by real-time polymerase chain reaction (PCR). **b** DDIT4 expression in GC tissues and paired normal tissues was determined by western blotting. **c**, **d** Immunohistochemistry revealed DDIT4 upregulation in GC tissues compared with normal tissues. *DDIT4* DNA damage-inducible transcript 4, *GC* gastric cancer, *N* normal tissue, *T* tumor tissue. ***P < 0.001, **P < 0.01, *P < 0.05

To determine the expression pattern of DDIT4 in GC, we measured DDIT4 expression in 20 pairs of GC and adjacent normal tissue specimens using real-time PCR and western blotting. DDIT4 expression was upregulated in 13 of 20 GC tissues compared with matched adjacent normal tissues (Fig. 1a). Similar to *DDIT4* mRNA levels, DDIT4 protein levels were increased in GC samples compared with their normal counterparts (Fig. 1b). Furthermore, we performed IHC using a GC tissue microarray containing 90 pairs of primary GC tissues and paired adjacent normal tissues. The IHC analysis revealed clear elevation of DDIT4 levels in GC tissues compared with the corresponding normal tissues, and DDIT4 expression was primarily located in the cytoplasm (Fig. 1c, d). Univariate survival analysis demonstrated that tumor size (*P *= 0.013), invasion depth (*P *= 0.015), lymphatic metastasis (*P *= 0.018), distant metastasis (*P *< 0.001), AJCC stage (*P *< 0.001), and pathological grade (*P *< 0.001) exhibit statistically significant associations with GC patient survival (Table 1). Taken together, DDIT4 expression was increased in GC tissues.

**Table 1:** Univariate and multivariate survival analysis of 90 GC patients

Variable	Univariate log rank survival analysis			Multivariate Cox survival analysis		
	RR	95% CI	*P* value	RR	95% CI	*P* value
Age (years)						
< 60	1.000					
≥ 60	1.085	0.832–1.413	0.547			
Gender						
Male	1.000					
Female	1.091	0.797–1.494	0.588			
Tumor size (cm)						
< 5	1.000					
≥ 5	1.182	1.036–1.349	0.013	1.387	0.827–2.325	0.215
Invasion depth						
T1/2	1.000					
T3/4	1.610	1.098–2.360	0.015	1.496	0.696–3.216	0.302
Lymphatic metastasis						
N0	1.000					
N1–3	1.369	1.056–1.774	0.018	1.093	0.456–2.621	0.842
Distant metastasis						
M0	1.000					
M1	1.468	1.268–1.299	< 0.001	4.364	1.830–10.409	0.001
AJCC stage						
I/II	1.000					
III/IV	1.500	1.214–1.853	< 0.001	1.755	0.768–4.011	0.183
Pathological grade						
I/II	1.000					
III/IV	1.912	1.125–3.250	0.017	2.077	0.863–4.996	0.103
DDIT4 expression						
Low	1.000					
High	3.583	2.345–4.271	0.146			

*RR* risk ratio, *95% CI* 95% confidence interval, *AJCC* American Joint Committee on Cancer

### Increased DDIT4 in GC cells promotes proliferation and colony formation

**Fig. 2:**
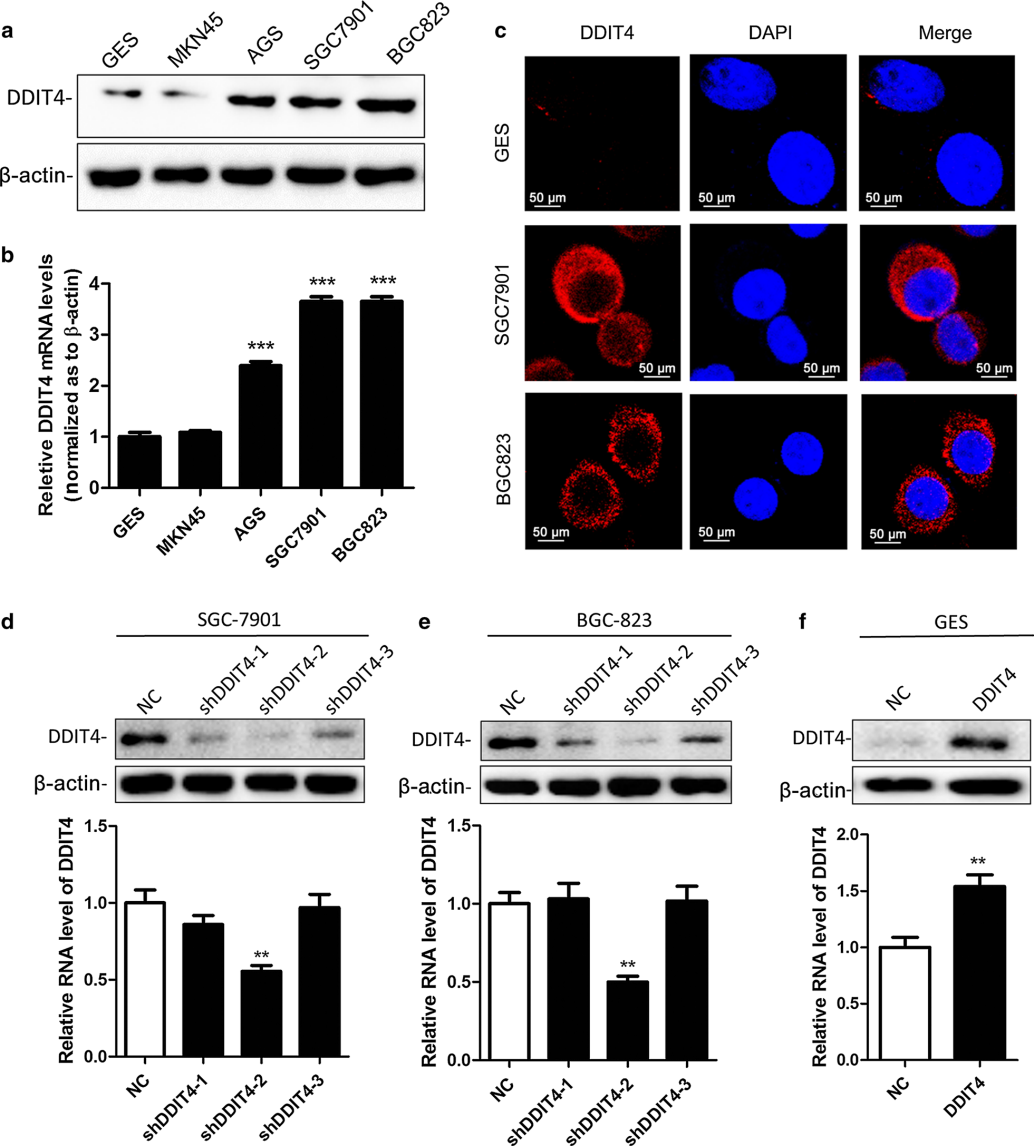
DDIT4 expression in GC cell lines. **a**, **b** DDIT4 protein and mRNA expression in the normal gastric cell line GES and in the GC cell lines MKN45, AGS, SGC7901, and BGC823. **c** The expression and subcellular localization of DDIT4 in GES, SGC7901, and BGC823 cells were examined by immunofluorescence. **d**, **e** Western blot and qRT-PCR analysis of DDIT4 expression in *DDIT4*-depleted SGC7901 and BGC823 cells. **f** Western blot and qRT-PCR analysis of DDIT4 expression in *DDIT4*-overexpressing GES cells. ***P < 0.001, **P < 0.01, *P < 0.05

To validate the expression pattern of DDIT4, we detected DDIT4 protein and mRNA levels in four GC cell lines (MKN45, AGS, SGC7901, and BGC823) and an immortalized gastric epithelial cell line, GES. Similar to the GC tissues, DDIT4 levels were significantly increased at both the protein and mRNA level in GC cells compared with GES cells (Fig. 2a, b). Moreover, immunofluorescence revealed that DDIT4 was mainly localized to the cytoplasm (Fig. 2c). To analyze biological function, we silenced *DDIT4* in SGC7901 and BGC823 cells with a lentiviral vector and upregulated *DDIT4* levels in GES cells using a *DDIT4*-overexpressing lentiviral vector. After cell transfection and antibiotic screening for 6 weeks, the lentiviral transfection efficiency was confirmed by real-time PCR and western blotting. Among three shDDIT4 vectors, shDDIT4-2 was the most effective at silencing *DDIT4* in SGC7901 and BGC823 cells (Fig. 2d, e). In contrast, transfection of the *DDIT4*-overexpressing lentiviral vector significantly upregulated DDIT4 levels in GES cells (Fig. 2f). Therefore, shDDIT4-2 and the *DDIT4*-overexpressing lentiviral vector were employed in the subsequent experiments.

**Fig. 3:**
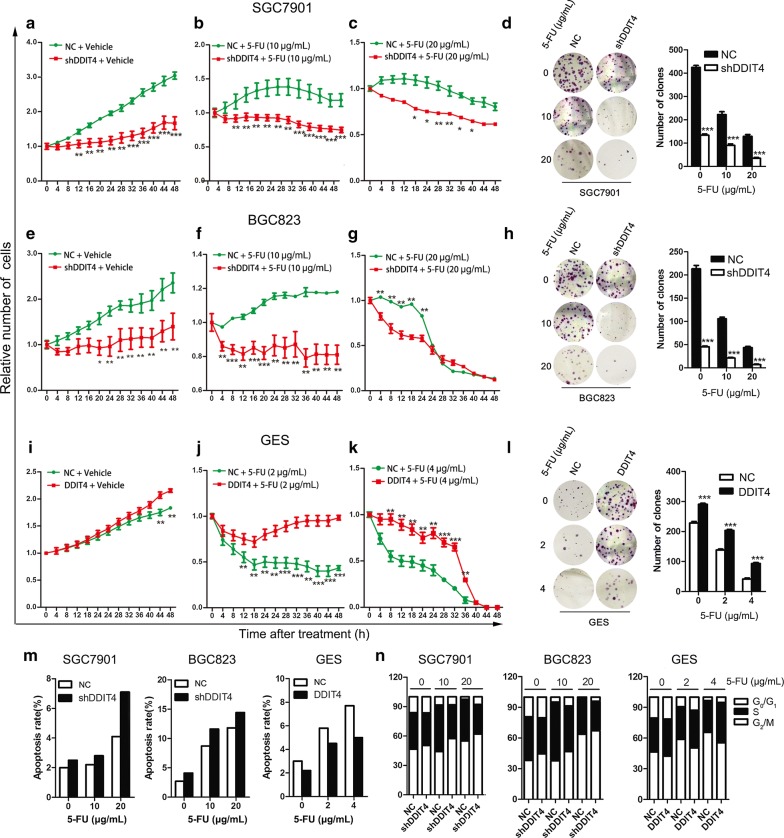
Effect of *DDIT4* silencing and overexpression on GC cell proliferation, apoptosis and cell cycle progression in vitro. **a**–**c** SGC7901 cell proliferation rate in response to 0, 10, and 20 µg/mL 5-FU was determined by high-content proliferation assays at various time points. **d** Representative images of SGC7901 cell colony formation after treatment with 0, 10, or 20 µg/mL 5-FU. The number of colonies was calculated, and the results are depicted in the bar chart. **e**–**g** BGC823 cell proliferation rate. **h** BGC823 cell colony formation assay. **i**–**k** GES cell proliferation rate. **l** GES cell colony formation assay. **m** Apoptosis in SGC7901, BGC823, and GES cells in response to 0, 10, or 20 µg/mL 5-FU as determined by flow cytometry. **n** Cell cycle distribution of SGC7901, BGC823, and GES cells treated with 0, 10, or 20 µg/mL 5-FU. ***P < 0.001, **P < 0.01, *P < 0.05

Given that DDIT4 has been implicated in oncogene- and stress-induced DNA damage [4], we hypothesized that DDIT4 upregulation might be involved in the initiation and development of GC. To test this hypothesis, we conducted high-content screening assays and colony formation assays to determine whether DDIT4 regulates GC cell proliferation. The high-content screening assays revealed that cell proliferation was significantly inhibited by *DDIT4* silencing in SGC7901 cells compared with the lentiviral control (Fig. 3a). Given that chemotherapeutics cause cytotoxic effects and DNA damage, we assessed whether DDIT4 was involved in the response of GC cells to these agents. Thus, we monitored SGC7901 cell proliferation after treatment with 5-FU (10 or 20 µg/mL) and found that *DDIT4* downregulation suppressed proliferation in the presence of 5-FU (Fig. 3b, c). Consistently, colony formation assays revealed that *DDIT4* downregulation inhibited SGC7901 colony formation with or without 5-FU (Fig. 3d). Similar to SGC7901 cells, *DDIT4* downregulation in BGC823 cells reduced cell proliferation (Fig. 3e–g) and increased sensitivity to 5-FU (Fig. 3h). In contrast, ectopic expression of *DDIT4* promoted GES cell proliferation and colony formation (Fig. 3i, l) and attenuated the sensitivity of GES cells to 5-FU (Fig. 3j–l). In addition, we performed Transwell assays to determine whether DDIT4 regulates GC cell migration and invasion, but no significant difference was observed in cells transfected with shDDIT4 lentivirus compared with the control group (Additional file 1: Figure S1). Taken together, these observations indicated that *DDIT4* acts as an oncogene that promotes GC cell proliferation and reduces chemosensitivity.

### Downregulation of *DDIT4* increases 5-FU-induced apoptosis but decreases 5-FU-induced S phase arrest in GC cells

Beneath the complexity and idiopathy of cancer lies a limited number of critical events that propel the tumor cell and its progeny into uncontrolled expansion and invasion [13]. Two such events are deregulation of apoptosis and the cell cycle, which together with the obligatory compensatory dysregulation of proliferation provide a minimal “platform” necessary to support further neoplastic progression [13]. Thus, we performed flow cytometry to determine whether DDIT4 modulates apoptosis and the cell cycle, which contribute to gastric carcinogenesis. *DDIT4* downregulation promoted apoptosis of SGC7901 cells treated with 0, 10 or 20 µg/mL 5-FU compared with the negative controls (Fig. 3m). In addition, *DDIT4* silencing in SGC7901 cells attenuated gastric cancer cell S phase arrest (Fig. 3n). Similarly, in BGC823 cells, *DDIT4* downregulation significantly increased GC cell apoptosis and reduced S phase arrest compared with the control (Fig. 3m, n). In contrast, *DDIT4* overexpression in GES cells significantly reduced apoptosis in the absence and presence of 5-FU (10 or 20 µg/mL) (Fig. 3m). Moreover, as demonstrated by cell cycle analysis, ectopic expression of *DDIT4* in GES cells drove the cell cycle into S phase and G_2_/M phase and reduced the population of cells in G_1_ phase compared with the control (Fig. 3n). Taken together, these findings indicated that DDIT4 is associated with 5-FU-induced apoptosis and cell cycle progression in GC cells in a dose-dependent manner.

### *DDIT4* silencing inhibits GC cell tumorigenesis in vivo

**Fig. 4:**
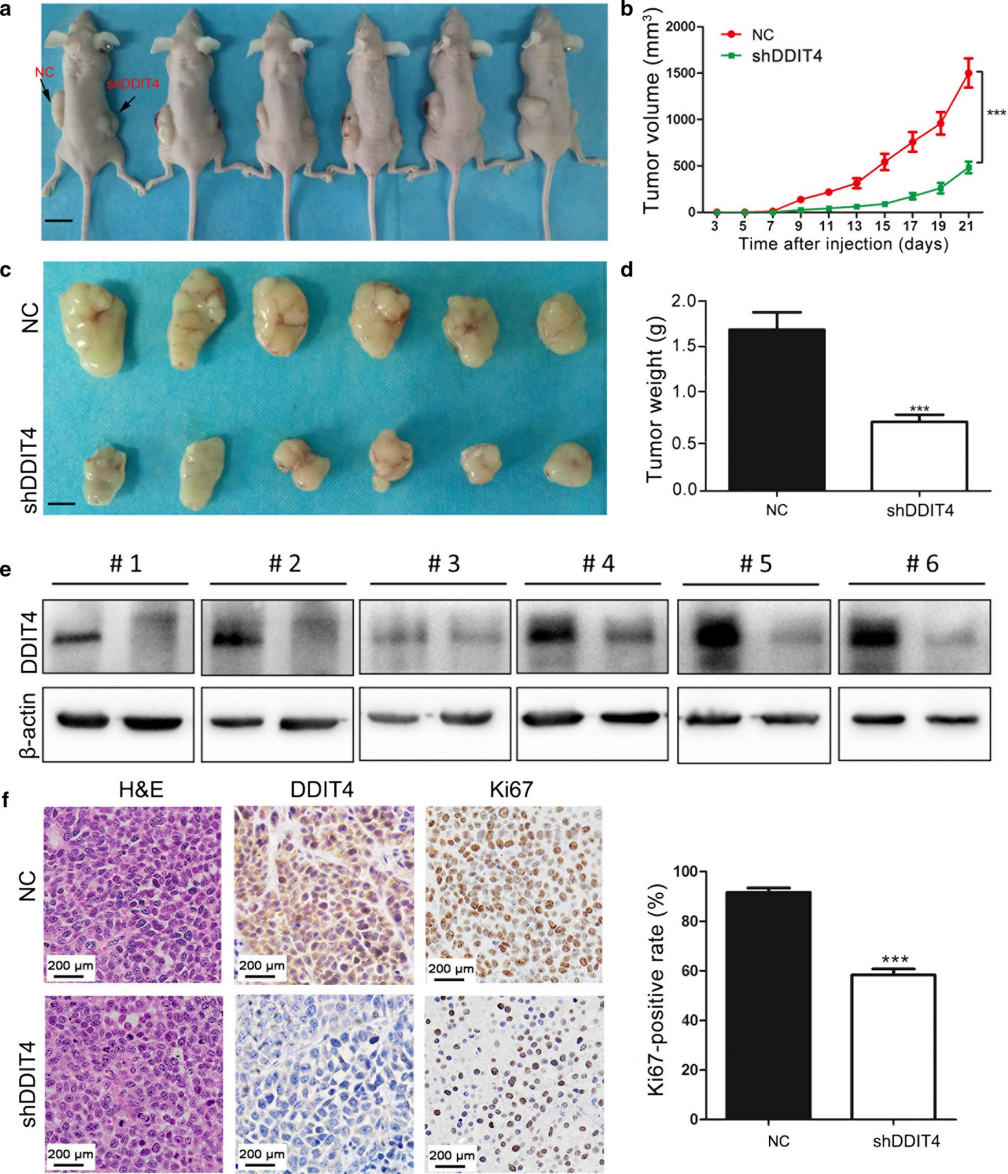
*DDIT4* depletion inhibited GC xenograft tumor growth in vivo. **a** BGC823 cells stably expressing si-DDIT4 or scrambled control siRNA were subcutaneously injected into nude mice. *DDIT4*-silenced BGC823 cells formed smaller tumors compared with cells expressing the scrambled control after 4 weeks. **b** Tumor growth curves. **c** Representative images of xenograft tumors. **d** Tumor weight. **e** DDIT4 expression in xenograft tumors was determined by western blot. **f** HE and immunohistochemical staining for DDIT4 and Ki67 in xenograft tumors. ***P < 0.001, **P < 0.01, *P < 0.05

To investigate the effect of DDIT4 on GC tumorigenic behavior in vivo, we conducted tumorigenicity assays in nude mice by subcutaneously injecting BGC823 cells stably expressing shDDIT4 or scrambled control shRNA into the dorsal flank of several mice. *DDIT4* depletion resulted in a significant reduction in tumor growth (Fig. 4a). *DDIT4*-knockdown tumors grew significantly slower (Fig. 4b) and weighed significantly less on average (Fig. 4c, d) compared with control tumors. Furthermore, we used western blotting and IHC to detect DDIT4 expression in xenografts and validated the lower DDIT4 levels in *DDIT4*-knockdown tumors compared with control tumors (Fig. 4e, f). Finally, we observed a reduced Ki67 (proliferation marker)-positivity rate in tumor tissues in the *DDIT4*-knockdown group compared with the control group (Fig. 4f). Taken together, these observations indicated that DDIT4 promotes GC growth in vivo and might function as an oncogene in gastric carcinogenesis.

### *DDIT4* downregulation inhibits GC cell proliferation through the MAPK and p53 pathways

**Fig. 5:**
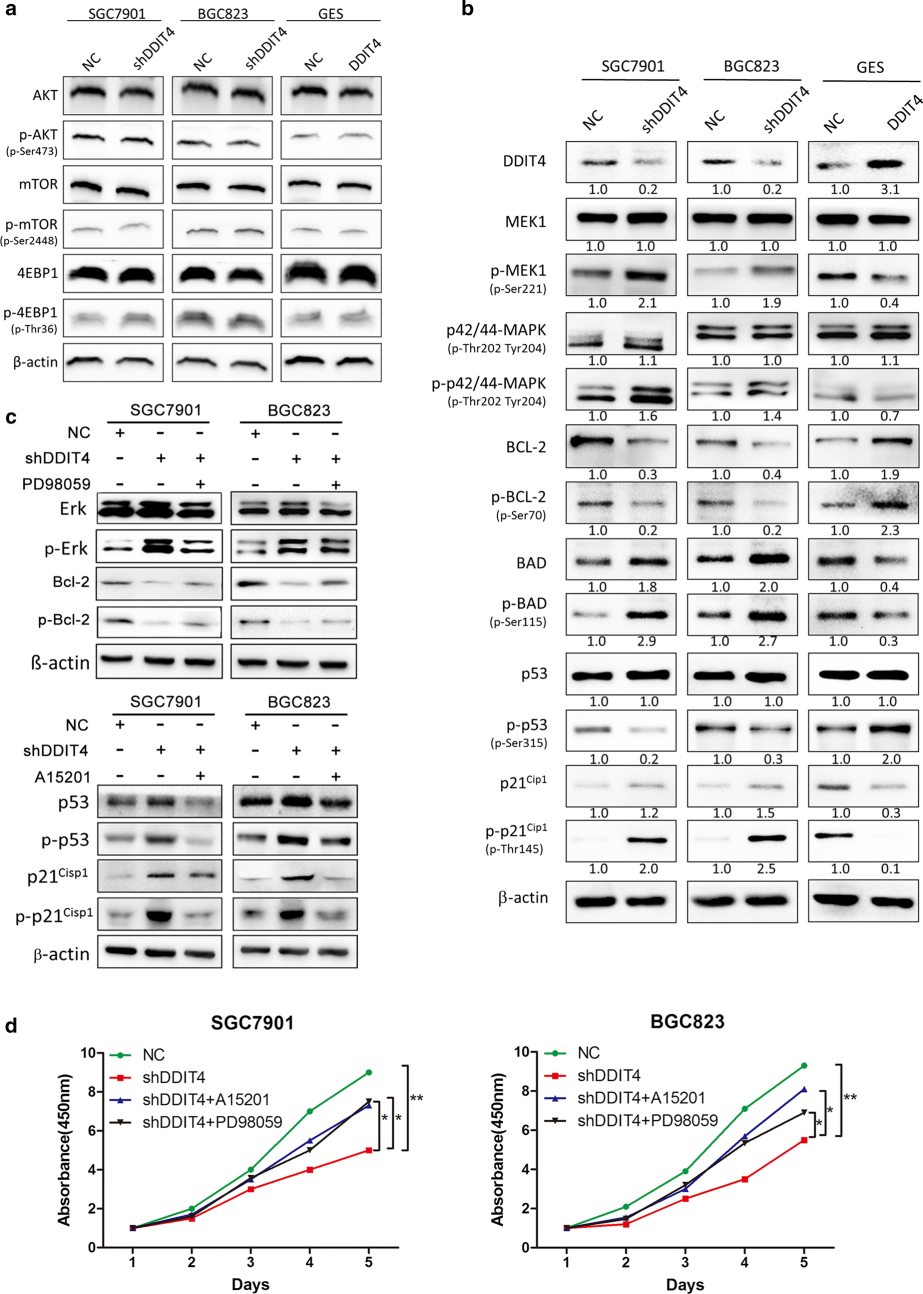
Downregulation of *DDIT4* activates the mitogen-activated protein kinase (MAPK) and p53 pathways in GC cells. **a** The total and phosphorylated levels of AKT, mTOR and 4EBP1 in *DDIT4*-silenced and *DDIT4*-overexpressing cells were examined by western blot. **b** The total and phosphorylated levels of MEK1, P42/44-MAPK, BCL-1, BAD, p53, and p21^Cip1^ in *DDIT4*-silenced and *DDIT4*-overexpressing cells were examined by western blot. **c**, **d** SGC7901 and BGC823 cells were infected shDDIT4 lentivirus and then were treated with inhibitors specific to p53 (A15201) or MAPK (PD98059). **c** The protein expression levels of phosphorylated and total ERK, BCL-2, p53 and p21^Cip1^ were analyzed by western blot. **d** Cell proliferation was analyzed by CCK8 assay

To understand the molecular mechanism underlying GC growth regulation by DDIT4, we utilized phospho-array assays that detect 131 phosphorylation sites in 12 critical cancer signaling molecules (Additional file 2: Table S1). Given that DDIT4 acts as a negative regulator of mTOR, we first examined the activity of the phosphatidylinositol-4,5-bisphosphate 3-kinase (PI3K)/AKTkt/mTOR pathway in GC cells upon *DDIT4* downregulation. Phospho-array assays revealed that the PI3K/AKT/mTOR pathway did not exhibit significant changes in *DDIT4*-knockdown cells (data not shown). Western blotting assays confirmed this observation (Fig. 5a), indicating that DDIT4 might activate other signaling pathways in GC cells. To uncover the molecular mechanism underlying GC growth regulation by DDIT4, we assessed MAPK and p53 signaling pathways in *DDIT4*-downregulated GC cells. In contrast to the PI3K/AKT/mTOR pathway, obvious alterations in the MAPK and p53 signaling pathways were observed (Fig. 5b). Anti-apoptotic BCL-2 levels were reduced, whereas pro-apoptotic BAD and proliferation-suppressive p21^Cip1^ levels were increased in *DDIT4*-downregulated cells (Fig. 5b). To further examine the effect of blocking the MAPK and p53 signaling pathways on gastric cancer cell proliferation following *DDIT4* knockdown, we performed rescue experiments by blocking the MAPK and p53 signaling pathways using a MAPK/ERK inhibitor (PD98059) and a p53 inhibitor (A15201) in *DDIT4*-knockdown cells. MAPK and p53 inhibition abolished the BCL-2 suppression and p21^Cip1^ elevation that was induced by *DDIT4* downregulation (Fig. 5c). Consistent with western blot assays, the CCK8 assays indicated that MAPK and p53 inhibition restored GC cell proliferation, which was suppressed by *DDIT4* knockdown (Fig. 5d). Taken together, these findings indicated that the MAPK and p53 signaling pathways might play a critical role in DDIT4-mediated GC cell proliferation.

## Discussion

Our study suggests that DDIT4 is an important regulator that is markedly upregulated in GC tissues and cells, and that knockdown of *DDIT4* suppresses the proliferation and tumorigenicity of GC cells both in vitro and in vivo. In addition, DDIT4 is attributed to chemotherapy-induced apoptosis and S phase arrest in GC cells. Mechanically, the proliferation-suppressive and chemosensitive effect of DDIT4 downregulation might be associated with activation of the p53 and MAPK signaling pathways.

GC currently poses a tremendous health burden on communities worldwide and is thought to result from a combined attack of environmental factors and genetic alterations [14, 15]. Among these factors, oncogene activation triggers replication stress and DNA damage, thereby increasing genome instability [16]. Additionally, the transient and long-term lack of nutrients, oxygen, and growth factors causes GC cells to be subject to frequent metabolic stress [17]. Thus, most GC cells display oncogene- or adverse environment-induced DNA damage [18]. DDIT4, a DNA damage-inducible transcript, is transcriptionally upregulated in multiple settings of DNA damage [4]. Notably, recent studies highlighted the important roles of DDIT4 in various types of human cancer [6, 19, 20]. In breast cancer, DDIT4 acts as a tumor-suppressor to regulate miR-495-mediated oncogenesis and hypoxia resistance [19]. Friedman et al. reported that DDIT4 enhances C/EBPbeta mediated autophagosome-lysosome fusion and desensitized PC cells to bortezomib [6]. In contrast, a positive correlation between DDIT4 and p-AKT was identified in ovarian cancer (OC), and DDIT4 expression in OC tissues was significantly increased in patients with serous adenocarcinoma and late FIGO stage [11], indicating that DDIT4 might be a tumor promotor in OC. These above findings demonstrated context-dependent regulation of DDIT4 in tumorigenesis and progression. However, whether and how DDIT4 plays critical roles in GC, which is characterized by frequent DNA damage, remains largely unknown. In the present study, we detected DDIT4 expression in GC tissues and cell lines, and found that DDIT4 was significantly upregulated in GC tissues and cell lines. In subsequent loss- and gain-of-function analyses, we observed that overexpression of *DDIT4* promoted GES cell proliferation, whereas knockdown of *DDIT4* suppressed GC cell proliferation both in vitro and in vivo. Therefore, our results demonstrated that DDIT4 is a proliferation-promoting and oncogenic protein in GC cells. Moreover, several lines of evidence demonstrate that DDIT4 is involved in anti-tumor chemotherapeutic treatment. For example, baicalein upregulates DDIT4 and causes mTORC1 and growth inhibition in platinum-resistant cancer cells [7]. Melatonin enhances arsenic trioxide-induced cell death via sustained upregulation of DDIT4 expression in breast cancer cells [21]. DDIT4 expression is an independent prognostic factor for triple-negative breast cancer resistant to neoadjuvant chemotherapy [12]. Here, we investigated the role of DDIT4 in response to increasing concentrations of 5-FU. We found that DDIT4 did not alter apoptosis and the cell cycle of GC cells in the absence of 5-FU but reduced apoptotic rate and S phase arrest in GES cells. In contrast, downregulation of *DDIT4* in GC cells increased cell apoptosis and S phase arrest. Collectively, our findings indicated that DDIT4 might contribute to GC development and chemosensitivity, suggesting that inhibiting DDIT4-mediated apoptosis and cell cycle arrest can lead to a greater apoptotic response and retard the cell cycle, thereby potentiating the efficacy of the chemotherapeutic agents against cancer.

Pinto et al. [12] demonstrated with an analysis in KM-Plotter that DDIT4 expression over the median is a protective factor for time to first progression (HR = 0.62; 95% CI 0.5–0.75, *P *= 1.7 × 10^−6^). However, analysis of the data downloaded from The Cancer Genome Atlas (TCGA) for gastric adenocarcinoma did not reveal differences in survival when comparing two groups with low and high DDIT4 expression (*P*-value in the log rank test of 0.999) [12]. In our study, we found that DDIT4 expression was increased in GC and that it functioned as an oncogene. In addition, univariate survival analysis revealed that DDIT4 did not exhibit statistically significant associations with GC patient survival, which is consistent with TCGA analysis for gastric adenocarcinoma. The inconsistencies of the evaluation of DDIT4 implied that the prognostic value of DDIT4 requires further investigation.

Reversible protein phosphorylation is one of the most important biological mechanisms for signal transduction, which is tightly regulated by protein kinases and phosphatases to maintain the balance of the protein’s phosphorylation status and control its biological functions [22]. Accumulating evidence indicates that perturbation of this balance contributes to the origin and pathogenesis of several human diseases. In cigarette smoke-induced pulmonary injury and emphysema, DDIT4 is necessary and sufficient for nuclear factor-kappaB (NF-kappaB) activation, and promoted alveolar inflammation, oxidative stress and apoptosis in alveolar septal cells [23]. DDIT4 promotes protein phosphatase 2A (PP2A)-dependent de-phosphorylation of AKT on Thr (308) but not on Ser (473) for phosphorylation of TSC2 [24]. Moreover, DDIT4 displays critical roles in hypoxia-inducible factor-1 (HIF-1) and p53 pathway crosstalk [4, 25]. However, the specific oncogenic pathway regulated by DDIT4 under different conditions remains unclear. In our study, we explored the mechanistic basis for DDIT4-mediated regulation of GC cells using phospho-antibody microarray-based proteomic analysis and found that the proliferation-suppressive and chemosensitive effect of *DDIT4* downregulation might be associated with activation of the MAPK signaling pathway, resulting in subsequent phosphorylation of BCL-2 (p-Ser70), which inhibits cell proliferation and induces apoptosis. Consistent with previous studies, we demonstrated that the MAPK pathway is frequently activated in human cancers, leading to malignant phenotypes such as autonomous cellular proliferation [26]. In addition to the MAPK signaling pathway, extensive studies have demonstrated that the p53 tumor suppressor protein preserves genome integrity by regulating growth arrest and apoptosis in response to DNA damage [27–29]. This notion is further supported by our data demonstrating that DDIT4 regulated the activation of multiple pro-apoptotic and growth-suppressive proteins, including p53. The p53 protein displayed a dual change in its phosphorylation state in *DDIT4*-knockdown cells compared with negative controls, as follows: (1) downregulation of phosphorylation at Ser6, which induces apoptosis; and (2) upregulation of phosphorylation at Ser315, leading to phosphorylation of p21^Cis1^ (p-Thr145), which rescues cells from apoptosis. Thus, in our study, we identified multiple pro-apoptotic and growth-suppressive proteins of which the phosphorylation and activation levels were regulated by DDIT4 in GC cells.

## Conclusions

In summary, our results demonstrated that DDIT4 promoted the tumorigenicity of gastric cancer cells by facilitating proliferation and colony formation and alleviating 5-FU-induced apoptosis through the p53 and MAPK pathways. The mouse model experiment further demonstrated that *DDIT4* downregulation significantly inhibited tumor growth in vivo. Taken together, our results suggest that DDIT4 may function as an oncogene in gastric cancer, providing a promising therapeutic strategy for GC treatment.

## Additional files

**Additional file 1: Figure S1.** The effect of DDIT4 downregulation on GC cell migration and invasion.

**Additional file 2: Table S1.** The effect of DDIT4-knockdown on phosphorylation of signal protein.

## Abbreviations

DDIT4: DNA-damage-inducible transcript 4
GC: gastric cancer
MAPK: mitogen-activated protein kinase
PC: prostate cancer
OC: ovarian cancer
NF-kappaB: nuclear factor-kappaB
PP2A: protein phosphatase 2A
