# Supplementary figures and images for: DDIT4 promotes gastric cancer proliferation and tumorigenesis through the p53 and MAPK pathways
Source: Cancer Commun (Lond). 2018 Jul 5;38:45. doi: 10.1186/s40880-018-0315-y (PMC6034313; doi:10.1186/s40880-018-0315-y)

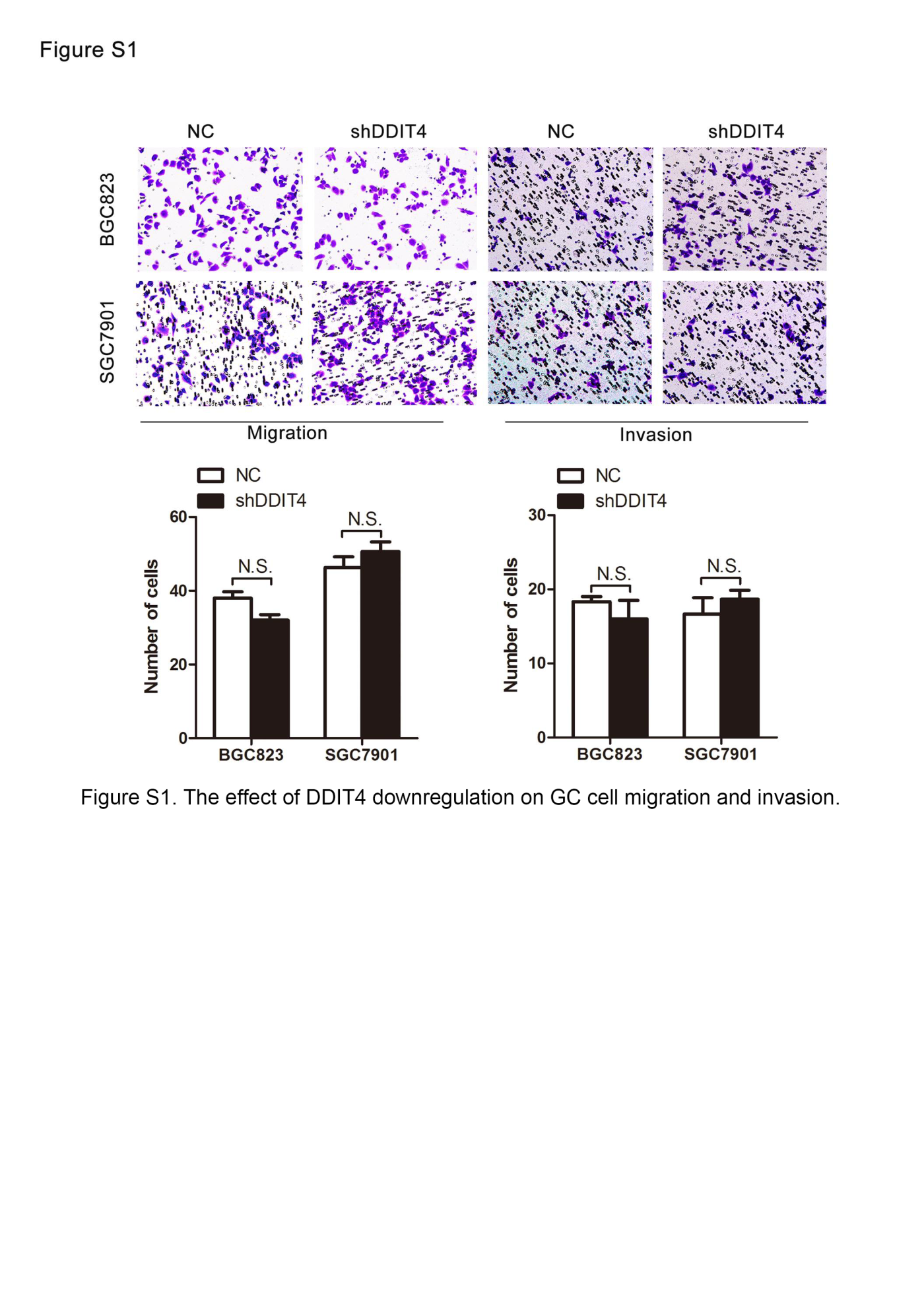

Supplement: Supplementary file 1 — Additional file 1: Figure S1. The effect of DDIT4 downregulation on GC cell migration and invasion. [file 40880_2018_315_MOESM1_ESM.docx]
